# Mutations Altering the Interplay between *Gk*DnaC Helicase and DNA Reveal an Insight into Helicase Unwinding

**DOI:** 10.1371/journal.pone.0029016

**Published:** 2011-12-13

**Authors:** Yu-Hua Lo, Shih-Wei Liu, Yuh-Ju Sun, Hung-Wen Li, Chwan-Deng Hsiao

**Affiliations:** 1 Institute of Molecular Biology, Academia Sinica, Taipei, Taiwan; 2 Institute of Bioinformatics and Structural Biology, National Tsing Hua University, Hsinchu, Taiwan; 3 Department of Chemistry, National Taiwan University, Taipei, Taiwan; Tulane University Health Sciences Center, United States of America

## Abstract

Replicative helicases are essential molecular machines that utilize energy derived from NTP hydrolysis to move along nucleic acids and to unwind double-stranded DNA (dsDNA). Our earlier crystal structure of the hexameric helicase from *Geobacillus kaustophilus HTA426* (*Gk*DnaC) in complex with single-stranded DNA (ssDNA) suggested several key residues responsible for DNA binding that likely play a role in DNA translocation during the unwinding process. Here, we demonstrated that the unwinding activities of mutants with substitutions at these key residues in *Gk*DnaC are 2**–**4-fold higher than that of wild-type protein. We also observed the faster unwinding velocities in these mutants using single-molecule experiments. A partial loss in the interaction of helicase with ssDNA leads to an enhancement in helicase efficiency, while their ATPase activities remain unchanged. In strong contrast, adding accessory proteins (DnaG or DnaI) to *Gk*DnaC helicase alters the ATPase, unwinding efficiency and the unwinding velocity of the helicase. It suggests that the unwinding velocity of helicase could be modulated by two different pathways, the efficiency of ATP hydrolysis or protein-DNA interaction.

## Introduction

DNA helicase is a motor protein that unwinds and separates duplex DNA using energy derived from the hydrolysis of nucleoside triphosphates (NTPs). This unwinding activity is ubiquitous in all pathways of DNA metabolism, such as DNA replication, repair and recombination [1], [2]. Helicases have been classified into six superfamilies (SF-I to SF-VI) based on conserved sequence motifs characteristic of proteins that catalyze directional translocation on nucleic acids [3], [4]. SF-I and SF-II helicases generally operate as monomers or dimers on a diverse range of DNA and RNA substrates, while SF-III to SF-VI helicases adopt hexameric structures and function in replication [4]. In general, the SF-IV hexameric helicases unwind DNA in the 5′ to 3′ direction. The unwinding of duplex DNA by the catalytic action of helicases involves unidirectional translocation and base-pair separation. Since all DNA substrates cannot be unwound within a single biochemical catalytic cycle, the helicase translocation along DNA must involve a series of cyclical binding and release events, which implies that the affinity of helicase for the DNA transiently changes during translocation [5].

Our earlier crystal structural studies of the *Gk*DnaC-ssDNA complex, identified ssDNA-binding pockets on the interior surface of the hexameric ring [6]. The ssDNA-binding pockets are directed toward the N-terminal domain collar of *Gk*DnaC, thus orienting the 5′-end ssDNA toward the DnaG primase to facilitate the synthesis of short RNA primers. We also have previously identified several critical residues that interact with the ssDNA [6]. The locations of these key residues imply that these ssDNA-interacting sites could correlate with helicase translocation. However, unidirectional translocation of the helicase along ssDNA may play a fundamental role in the unwinding process. To understand the hexameric helicase-catalyzed unwinding process in detail, we studied how the helicase and ATPase activities are correlated with the DNA binding. The approach taken is to acquire a detailed knowledge of the structure and the kinetic/mechanistic information. Recently, single-molecule (sm) techniques, such as total internal reflection fluorescence microscopy (TIRF), single-pair FRET, magnetic/optical tweezers, have been shown to provide novel mechanistic details in helicase action [7]. These single-molecule techniques directly detect the helicase translocation and unwinding in real-time, as well as the conformational dynamics in protein–DNA and protein–protein interactions during helicase action, offer new information that were averaged-out in typical ensemble biochemical experiments. For example, optical and magnetic tweezers-based single-molecule experiments have been used to study several ring-shape helicases, and have determined the force dependence of helicase unwinding kinetics [8], [9], [10]. These studies indicate that phage T7 gp4 helicase unwinds DNA in an active form [8], whereas the phage T4 gp41 helicase unwinds DNA passively [9]. *E. coli* DnaB helicase was shown by magnetic tweezers experiments that its unwinding rate, translocation rate, and pausing activity can be modulated by force as well as DNA geometry [10]. In addition, complex dynamics in replication fork, such as T4 primosome, was also studied by single-molecule methods to show the coordination among different protein complex during replication [11].

In this study, we use the replicative helicase of *Geobacillus kaustophilus*, a gram-positive bacterium, as a model system (*Gk*DnaC). To investigate the nature of unwinding by hexameric replicative *Gk*DnaC helicase, we studied how the activity of *Gk*DnaC is modulated by specific mutations and by accessory proteins. We first compared the unwinding efficiency of these helicases, using gel shift assays, and measured their ATPase activities using spectrophotometric assay. We also developed a single-molecule tethered particle motion (smTPM) experiment to directly determine the unwinding velocity of replicative helicase *Gk*DnaC in real-time. The observation by smTPM had several advantages for this study. First, the method is suited for length and elasticity study of the DNA molecules, which are correlated to the helicase-mediated unwinding processes. Second, the technique allows the study of helicases interaction on torsion relaxed DNA molecules in a nearly force-free experiment. Surprisingly, weakened interaction between helicase and ssDNA leads to an enhancement in helicase efficiency and faster unwinding velocity, while ATPase activities remain unchanged. In strong contrast, adding accessory proteins (DnaG or DnaI) to *Gk*DnaC helicase, alters the ATPase, as well as the unwinding efficiency and the unwinding velocity of the helicase.

## Materials and Methods

### Cloning, expression and purification

Protein expression and purification of *Gk*DnaC wild-type (WT) and mutants were done as previously described [6]. For control experiments, the new constructs *Gk*DnaC K309A and R420A mutants were generated according to the QuikChange mutagenesis protocol (Stratagene, La Jolla, CA) using the pET21b-*Gk*DnaC wild-type plasmid as the template. These mutants were overexpressed and purified similar to WT, and showed identical chromatographic behavior as that of the *Gk*DnaC WT on a size-exclusion column (data not shown). Therefore, amino acids substituted on these mutants do not affect hexameric formation. The coding region of full-length *Gk*DnaG was generated by PCR amplification of genomic DNA isolated from *Geobacillus Kaustophilus* HTA426 using *Pfu* DNA polymerase (Stratagene). The forward and reverse primers were designed to incorporate unique NdeI and XhoI restriction sites, respectively, permitting the insertion of the amplified product into the pET21b vector (Novagen) for protein expression in *E. coli*. The resulting plasmid, pET21b-*Gk*DnaG, encodes full-length wild-type *Gk*DnaG fused with a C-terminal His_6_ tag (LEHHHHHH). *Escherichia coli* BL21(DE3) cells (Yeastern Biotech. Co., Ltd.) were transformed with these expression vectors and grown at 37°C in Luria-Bertani medium containing 50 µg/ml ampicillin until the OD_600_ reached a value of 0.7. Overexpression of *Gk*DnaG was induced with 1 mM IPTG for 6 h at 20°C. Harvested cells were resuspended in buffer A (10 mM sodium phosphate, pH 7.0) and then lysed by sonication. Due to the low binding affinity by His-trap column, *Gk*DnaG proteins were purified from the soluble supernatant on a HiTrap Heparin HP column (5×5 ml, GE Healthcare) followed by purification on a Q-sepharose column (GE Healthcare). The purified proteins were collected and dialyzed against buffer B (10 mM Tris-HCl, pH 8.0, 100 mM NaCl). Column fractions were analyzed by SDS-PAGE. Details for construction and protein purification of co-expressed *Gk*DnaC-*Gk*DnaI complex have been described previously [12].

### ATPase assay

The ATPase assay is based on a reaction in which ATP hydrolysis is coupled to the NADH oxidation. The kinetics of NADH disappearance was monitored at 340 nm using a spectrophotometer (Shimadzu UV1800). The assay was performed at room temperature with a reaction buffer containing 50 mM Tris-HCl (pH 7.4), 0.8 mM DTT, 20 mM β-mercaptoethanol, 0.5 mg/ml BSA and 5 mM MgCl_2_. The reaction mixture was supplemented with 11.36 U/ml phosphoenolpyruvate (PEP), 20 U/ml pyruvate kinase (PK), 20 U/ml L-lactate dehydrogenase (LDH), 0.08 mg/ml NADH, 5 mM ATP, and 1 µM *Gk*DnaC helicase (or *Gk*DnaC mutants) in the absence or presence of 15-mer single-stranded oligo(dT) (50 nM) in a final volume of 150 µl. All chemicals were purchased from Sigma. Experiments using longer DNA (30- and 70-nt) substrates yielded similar ATPase rates (Figure S1). The rate of ATP hydrolysis is proportional to the rate of the decrease in absorbance at 340 nm and is calculated according to the formula: ΔA_340_/time (s^−1^) ×9820 =  rate of ATPase (µM/min) [13].

### Gel shift assay

A 60-nt-long oligonucleotide (5′-ACATGATAAG ATACATGGAT GAGTTTGGAC AAACCACAAC GTAAAACGAC GGCCAGTGCC-3′, Mission Biotech) at 1 mM was biotin-labeled at the 3′ end using terminal deoxynucleotidyl transferase (TdT, Thermo) and then annealed to an equal molar ratio of M13mp18 single-stranded circular DNA (NEW ENGLAND, BioLabs). M13mp18 vector is derivative of the single-stranded, male-specific filamentous DNA bacteriophage M13 and it is 7249 bp in length. The mixture was heated at 95°C for 2 min, then allowed to anneal at 65°C for 20 min, and cooled down slowly to room temperature. The 3′-terminal region (20 nt) of the oligonucleotide is complementary to M13mp18 ssDNA, while the remaining region forms a long overhang tail to create a replication fork-like template. The DNA substrates were purified on a Sepharose CL-4B spin column according to the protocol for separation and isolation of small and large DNA fragments [14]. DNA-unwinding activity assays with *Gk*DnaC helicase were carried out using an ECL-EMSA kit (enhanced chemiluminescence electrophoretic mobility shift assay, Thermo). Approximately 125 nM of the fork-DNA substrate was incubated at 37°C for 40 min with the *Gk*DnaC proteins (1 µM refer to monomer) in 50 mM Tris-HCl (pH 7.4) buffer containing 20 mM β-mercaptoethanol, 5 mM MgCl_2_, 5 mM ATP and 0.5 mg/ml bovine serum albumin. The reaction was terminated by adding 5 µl of 5X stop solution containing 0.04% SDS, 8% glycerol and 40 mM EDTA (pH 8.0). The amount of junction dissociation was analyzed by electrophoresis using a 10% native polyacrylamide gel run in 0.5x TBE for 2 h at 100 V. The biotin-labeled DNA was then transferred to a positive nylon membrane, UV cross-linked, probed with streptavidin-HRP (horseradish peroxidase) conjugate and incubated with the chemiluminescent substrate. The membrane was then exposed to X-ray film for quantification in an AlphaImager 2200 gel documentation system. For all reactions, unwinding efficiency was defined as the fraction of the unwound ssDNA fragment over all biotin-labeled signals. To normalize these values, the percentage of product was calculated using the equation, %Unwound  = (%U_S_–%U_0_)/(%U_100°C_–%U_0_) where %Us represents the percentage unwound in the sample lane of interest, %U_0_ is the percentage unwound in the unreacted substrate and %U_100°C_ is the percentage unwound in substrate treated at 100°C. Each experiment was performed at least in triplicate. This gel shift assay compares the helicase unwinding efficiency by quantifying the amount of DNA unwound at a given time interval for wild-type and mutant helicases.

### Single-molecule TPM measurements: DNA substrates

In order to observe the enzyme unwinding process at the single-molecule level, we designed a fork DNA substrate called “fork-AC90”. The DNA substrate consists of three annealed oligonucleotides. Oligonucleotide A is a 145 nt TG-rich strand (5′-TTTTTTTTTT TTTTTCCAGT CACAGAAAAG CATCTTATGT GACCGTCTCT GTGTGCTGGT GGGTGTGTGT GTGCTGGTGG GTGTGTGTGT GCTGGTGGGT GTGTGTGTGT GTGTGTGCAG GTGTAGACTA CAGCGTGAGC TATGA, Sigma). This TG-rich strand A was then annealed to two other oligonucleotides to create a nicked substrate: one is an 18 nt-long, 5′-end digoxigenin-labeled oligonucleotide B (5′-dig-TCATAGCTCACGCTGTAG, Sigma), and the other is a 90 nt-long, 3′-end biotinylated oligonucleotide C carrying AC repeats (AC90). The annealed DNA substrate contains a 37 nt single-stranded overhang at its 5′-end for helicase loading and can be bound to streptavidin-coated beads (200 nm, Bangs Lab, Fishers, IN) through the biotin-labeled end for single-molecule tracking. The AC repeat sequence was designed to avoid potential secondary structure formed during the helicase unwinding. The annealed DNA substrates were purified from excess individual free oligonucleotides using a PCR purification kit (Qiagen). To mimic the unwound product, we also prepared another DNA substrate similar to fork-AC90 substrates except 108 nt from the 5′ end of oligonucleotide A was removed, leaving a 37 nt long oligonucleotide.

### Imaging acquisition and analysis

The Brownian motion (BM) of tethered beads was observed by means of an inverted optical microscope (Olympus IX-71, Tokyo, Japan), using a Newvicon camera (NC-70, DAGE-MTI, Michigan, IN) by the differential interference contrast (DIC) method at 30 Hz [15]. The images were directly digitalized through a frame-grabber (PCI-1411, National Instrument, Austin, Texas) and stored in a computer. For time-course studies, the amplitude of bead BM was quantified using the mean-squared displacement (MSD; <Δx^2^> = <x^2^>–<x>^2^) of drift-correlated centroid positions of the tether beads from 20 consecutive frames (0.66 s). For population distribution measurements, MSD values were calculated from 1000 consecutive frames to obtain the average BM amplitude. To ensure that only one DNA molecule was attached to each bead, only the beads with symmetrical BM were included in analysis, and the ratio of MSD values of x position and those of y position of the beads was limited to 1.1–0.9 [16], [17], [18].

### DnaC helicase unwinding experiments by smTPM

The preparation of the slide chamber and streptavidin-coated beads have been described previously [15]. The fork-AC90 DNA substrate was anchored on an anti-digoxigenin coverslip through its digoxigenin label. After 30 min incubation at room temperature, the unbound DNA was removed. Then streptavidin-coated polystyrene beads were flowed into the reaction chamber to attach onto the biotin group in the fork-AC90 substrate. After removal of excessive beads, *Gk*DnaC helicase was introduced to load onto the fork-AC90 substrates through its distal 5′-overhang in the presence of ATP to initiate unwinding. The 30 µl, 500 nM enzyme (*Gk*DnaC helicase, *Gk*DnaC-*Gk*DnaG complex, *Gk*DnaC-*Gk*DnaI complex and *Gk*DnaC mutants) in the reaction buffer (50 mM Tris-HCl, pH 7.4, 5 mM MgCl_2_, 2 mM β-mercaptoethanol, 5 mM ATP with PK-PEP regeneration system, 2 mg/ml BSA) was flowed into the slide chamber at 22±1°C. Further reduction of enzyme concentration greatly reduced the observed unwinding probability. Reactions using higher enzyme concentration (10 µM) returned the same unwinding velocity (data not shown). Thus, it is most likely that a single hexameric DnaC unwinds the DNA substrate at this concentration. The DNA substrate in the unwinding experiment was designed so that the dissociation of the bead-labeled oligonucleotide AC90 signals the completion of unwinding of the 90 bp duplex region. During the unwinding of the 90 bp duplex substrates, the conversion of duplex into single strands lead to an increase in BM, as confirmed by the BM increase from the fork-AC90 substrate to the mimicked unwound substrate. The Gaussian fitting of the forked (N = 66) and mimicked unwound (N = 57) substrates returned with a mean±standard deviation of the distribution. The change in the BM of the beads thus correlates with the *Gk*DnaC-mediated unwinding kinetics. The conversion factor is based on the difference BM between these two substrates and their difference on duplex length, and is determined to be 0.0718±0.0257 nm/bp. Only the beads with increasing BM pattern prior to dissociation are included in unwinding velocity analysis. Since the tethers appeared in the absence of *Gk*DnaC, we identified the time point where *Gk*DnaC binds and the unwinding starts by the changing point in the first derivative of the BM time-course (as shown in Figure S2). Once the initial unwinding time point is identified, the unwinding velocities were determined by applying least-squares linear fits to the increasing BM section of the BM time-courses. The unwinding velocities were also determined using the dwell time between the beginning of BM increase and the maximum BM achieved, required to unwind the 90 bp duplex DNA. Both methods yielded the same unwinding velocities within experimental errors.

### Bootstrap estimation for Unwinding velocities

The histograms of mean unwinding velocities were supplied by bootstrapping estimation that resamples the means of the unwinding velocities 100 times. The bootstrapping estimation was executed by R-software (http://www.r-project.org/). We used the ordinary nonparametric bootstrapping method to generate the t-distributions of unwinding velocities based on 10–18 smTPM measurements.

## Results and Discussion

### Helicase activity alters as helicase loses partial DNA-binding interaction

**Figure 1:**
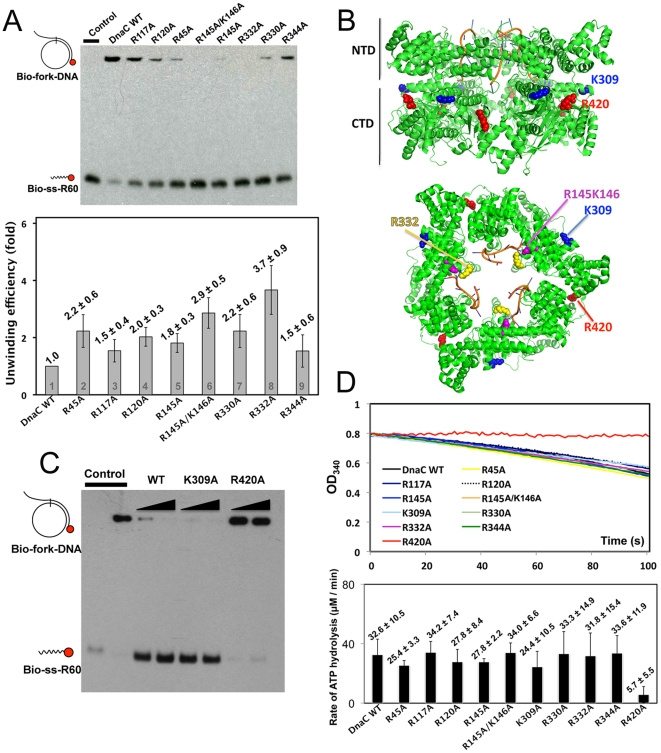
Ensemble assays of wild-type and mutant *Gk*DnaC. A. Gel shift assay of *Gk*DnaC WT and mutants. The enhancement of unwinding efficiency is expressed by the fold of change in the biotin-labeled ssDNA unwound product from *Gk*DnaC mutant to that from WT. Fork-DNA substrate and ssDNA product are indicated schematically on the left. The relative amount of unwinding efficiency of each mutant with reference to *Gk*DnaC WT is indicated. The red sphere denotes the biotin-labeled end. The average and standard deviations of three independent experiments are given. **B.** Locations of amino acid residues in *Gk*DnaC that were mutated. The hexameric *Gk*DnaC and ssDNA are represented as cartoons and colored in green and orange, respectively. **C.** Gel shift assay of *Gk*DnaC WT, mutant K309A and R420A (arginine finger). An increasing amount of purified protein (0.5 to 1 µM) was used in the standard gel shift assay. **D.** Time-courses of ATPase activity of *Gk*DnaC WT and mutants. Reactions were initiated by the addition of 1 µM enzyme (per monomer) after preincubation of all other components at RT for 5 min. ATPase rates of WT and mutants are compared. Data represent the average and standard deviations from three independent experiments.

Based on the crystal structure of the *Gk*DnaC-ssDNA complex and biochemical studies on the mutant proteins, we have previously identified several key residues of *Gk*DnaC involved in ssDNA binding [6]. Previous studies showed that mutations of residues located within the inside channel of the helicase and involved in ssDNA-binding (i.e., K50A, R117A, R120A, R145A, R145A/K146A, R330A, R332A and R344A) increased the dissociation constants of the resulting enzymes by two to three orders of magnitude as compared to that of the wild-type *Gk*DnaC helicase [6]. We also presented the sequence alignment for these residues in the ssDNA-binding region of *Gk*DnaC helicase among DnaB-like family, as shown in Figure S3. According to the alignment analysis, some residues are completely conserved among family members (such as R117, R344 and R420), while others belong to a nearly identical or similar group (R45, R120, R145, K146, K309, R330 and R332). We thus suggest that these residues that located at the central channel of helicase play key roles for DNA-binding among DnaB-like family members, which in turn can affect DNA translocation during the unwinding process. To test if these mutations indeed alter helicase unwinding, we analyzed the *Gk*DnaC helicase unwinding efficiency by monitoring the amounts of the helicase-catalyzed displacement of short biotin-labeled oligonucleotides from the forked DNA substrates. We first compared the amount of unwinding products generated by wild-type and mutant helicases within a given reaction time using gel shift assay. To optimize experimental sensitivity for the gel shift assay, we first identified the unwinding product as a function of enzyme concentration in a fixed reaction time of 1 hour at the constant amount of DNA substrates. Using increasing amounts of wild-type *Gk*DnaC WT (0.1 to 4 µM), we found that the relative unwinding efficiency was essentially proportional to the amount of enzyme and displayed a linear gradient up to 1 µM, and reached the plateau when enzyme concentration is higher than 1 µM (Figure S4). We then carried out the gel shift assay for monitor the unwinding efficiency of wild-type and mutant helicases at 1 µM protein concentration at 37°C for slightly shorter time of 40 minutes (Figure 1A). Interestingly, most *Gk*DnaC mutants in which a positively charged side chain within the center channel had been replaced by an alanine residue showed a significantly enhanced helicase activity (Figure 1A). Among these mutants, the R332A mutant showed a nearly 4-fold increase in helicase unwinding efficiency, suggesting that the DNA-binding loop I (residues 321 to 335, shown in Figure 1B) located in the inside channel of the hexameric helicase is not only responsible for DNA binding but also affects significantly helicase unwinding and/or translocation. By contrast, mutation of a positively charged residue (K309A) located on the outside surface of the hexameric ring and away from the DNA-binding channel (Figure 1B) did not affect the unwinding efficiency of the enzyme (Figure 1C). As a control, mutation of the arginine (R420A) residue located in the nucleotide-binding pocket from the adjacent subunit rendered the enzyme completely inactive (Figure 1B and 1C). This control experiment indicated that the mutation in the ATP-binding site of *Gk*DnaC abolishes the helicase function. These results suggest that partial loss of the interaction between the helicase and ssDNA can alter the helicase unwinding activity. From the structural point of view, these key residues all located in central channel of helicase, we thus considered that protein-ssDNA interaction may correlate with the helicase translocation. Helicase that lost the partial interaction with DNA might translocate faster than before resulting in efficient unwinding. In this weak binding state, helicase could incompletely dissociate from the nucleic acid and easily move forward to the junction.

### ATPase activities of wild-type *Gk*DnaC and mutants

The unwinding activity of helicase requires its coupling with ATP hydrolysis. To understand if the enhanced helicase activity result from differences in ATPase rates of different helicase mutants, we measured the ATPase rates of wild-type *Gk*DnaC and its mutants. Measurements of ATP hydrolysis were performed both in the absence and presence of ssDNA. The DNA-independent ATPase activity provides information about the intrinsic ability of proteins to hydrolyze ATP, whereas the measurement of ATPase activity in the presence of ssDNA reflects the DNA-stimulated ATPase activity. We carried out a coupled ATPase spectrophotometric assay by monitoring the decrease of NADH absorbance at 340 nm (Figure 1D). As expected, the nucleotide-binding pocket mutant R420A showed a low ATPase rate of about 5.7 µM/min (red line, Figure 1D) in the absence of ssDNA, similar to background level of 3.5 µM/min (data not shown). However, for wild-type enzyme and helicase mutants with enhanced activities, the intrinsic ATPase rates are all about 30 µM/min. The similar intrinsic ATPase rates of wild-type and mutants indicated that the enhancement of helicase unwinding activity in mutant proteins is not due to an increased efficiency of ATP binding or ATP hydrolysis but resulted from the helicase/ssDNA interaction. On the other hand, the DNA-stimulated ATPase activities we observed here were only slightly increased as compared to the intrinsic ATPase activity. Although we have used different lengths or different concentrations of ssDNA, the measured ATPase rates are rather similar with the intrinsic ATPase rates (Figure S1 and S5). It is possible that DNA binding does not stimulate ATPase activity in *G. kaustophilus* hexameric DnaC helicase. Similar observations have been reported for several other hexameric helicases such as *Bst*DnaB, TWINKLE, and *Eco*RuvB helicases [19], [20], [21].

### Helicase unwinding velocity monitored by smTPM

**Figure 2:**
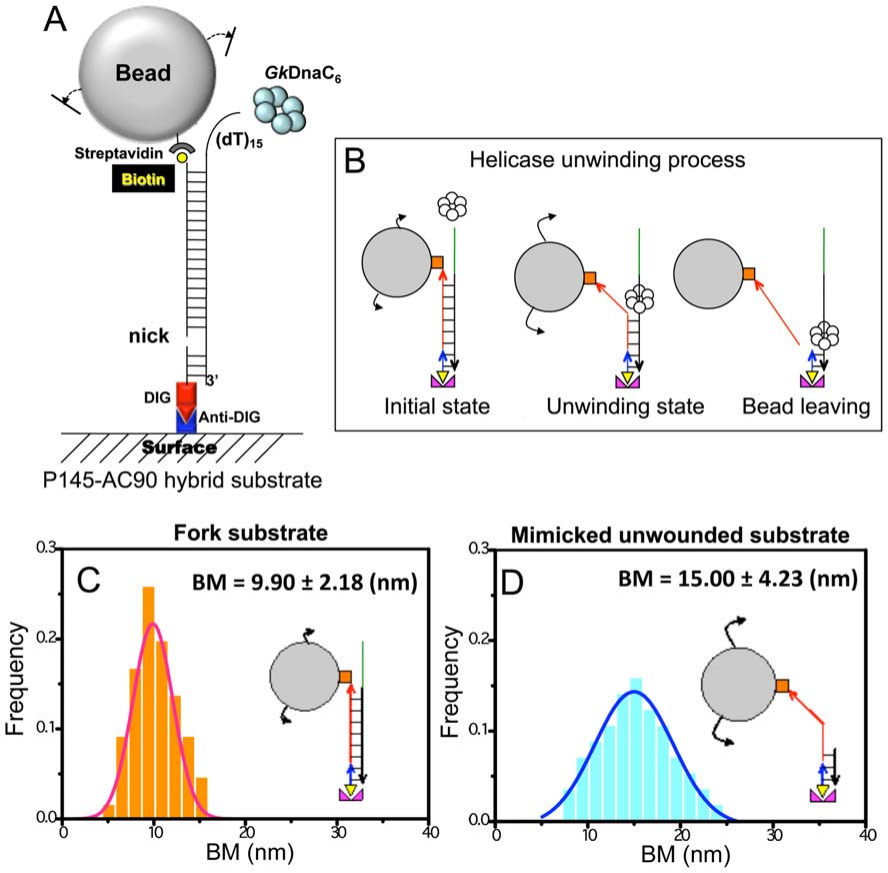
Single-molecule TPM to monitor *Gk*DnaC unwinding. **A.** Experimental geometry for monitor unwinding velocity. **B.** *Gk*DnaC-mediated unwinding leads to the conversion of duplex DNA into ssDNA, which is signaled by an increase in the BM of the bead, and eventually followed by bead-ssDNA disappearance. In the absence of helicase, a smaller, constant BM amplitude corresponding to the duplex substrate was observed. As *Gk*DnaC binds and initiates unwinding, BM starts to increases. Due to the design of the nicked substrate, the ssDNA-tethered bead disappears and signals the completion of unwinding of the 90 bp duplex. **C.** The initial BM distribution of the fork substrate with 108 bp duplex DNA and 37 nt ssDNA overhang allowing for *Gk*DnaC binding is 9.90±2.18 nm (mean±s.d., N = 66). **D.** The BM for the mimicked unwound product before bead disappearance. The substrate contains a 37 bp duplex region with a nick and a 71 nt ssDNA. The BM increases to 15.00±4.23 nm (mean±s.d., N = 57). It is feasible to monitor the helicase unwinding using the BM increase.

To test whether the differences in unwinding efficiencies between the *Gk*DnaC helicase and its mutants seen in gel shift assays result from the differences in unwinding velocities, we directly measured the unwinding velocities of individual *Gk*DnaC helicases in real-time at the single-molecule level. Most single-molecule studies on ring-shaped SF-IV helicases used force-dependent techniques such as optical and magnetic tweezers (in the force range of 5–40 pN) [7], [8], [9], [10], [22], [23]. Here, we developed a single-molecule tethered particle motion (smTPM) experiment to study the unwinding velocity of *Gk*DnaC helicase in a nearly force-free condition (in the force range of fN). We tethered the DNA substrates on anti-digoxigenin-decorated coverglass and labeled the distal 5′ end of DNA with streptavidin-coated beads (see Materials and Methods, Figure 2A). The centroid position of the tethered bead can be measured by optical microscopy and determined to nanometer precision by image processing [16]. As unwinding persists, duplex DNA is converted to ssDNA that possesses a higher flexibility. Therefore, a gradual increase in Brownian motion (BM) of the bead is expected from the unwound ssDNA (Figure 2B). The BM of intact, initial fork-AC90 substrates is shown to be fitted into a single Gaussian curve, 9.90±2.18 nm (mean±standard deviation; N = 66, where N is the number of effective tethers; Figure 2C). To mimic the unwound fork substrate before detachment, we replaced the 145 nt ssDNA with a 37 nt ssDNA annealed to the 3′-end of the biotinylated AC90 ssDNA. The BM of this sample was determined as 15.00±4.23 nm (mean±standard deviation, N = 57) as illustrated in Figure 2D. The increased BM reflects the difference between duplex DNA and ssDNA, and confirms that this BM increase can be used to monitor the DnaC-mediated unwinding process with the conversion factor of 0.0718±0.0257 nm/bp. The broader BM distribution of this unwound state is consistent with the long single-stranded DNA segment of this substrate.

**Figure 3:**
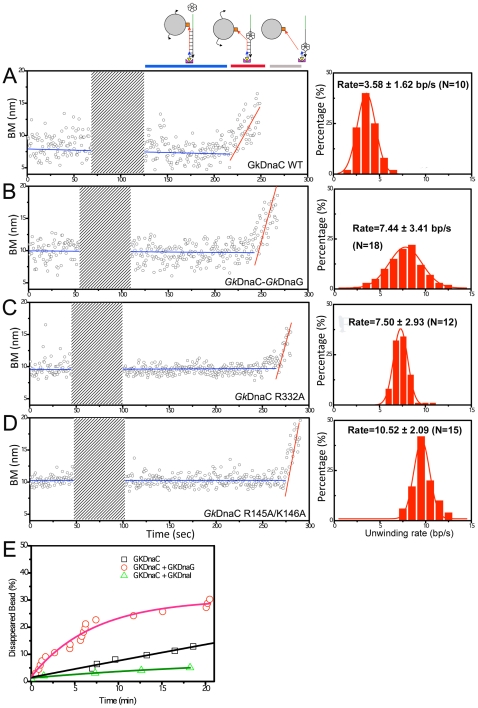
Observed unwinding by an individual *Gk*DnaC **enzyme in a smTPM experiment.** BM time-course of a single duplex DNA molecule in the presence of the indicated enzyme(s) (left panel) and the histogram of observed unwinding velocities (right panel). **A.** BM time-course of a single duplex DNA molecule unwound by *Gk*DnaC WT. There is a recording dead time of at least 20 s due to solution exchange and stage restabilization for imaging (shaded area). The increased slope of BM time-course was fitted to yield an unwinding velocity of 3.59±1.30 bp/s for this molecule. Histogram supplemented by data from bootstrapping statistics based on the mean of the measured unwinding velocities, shows the unwinding velocity (mean±s.d., N = 10). **B**–**D.** BM time-course for *Gk*DnaC-*Gk*DnaG complex, *Gk*DnaC mutant R332A and R145A/K146A. The unwinding velocities of individual traces are 7.33±2.63, 7.30±2.66, and 10.56±3.82 bp/s, respectively. The mean unwinding velocities are shown in the histogram. **E.** Time-course of disappeared tethered beads (in percent) observed by single-molecule TPM experiments. The cumulative exponential curve was fitted with the equation: *y = y_0_ + [1-(1/t)e^(−x/t)^]*.

Upon the addition of *Gk*DnaC helicase and the mixture of ATP and ATP regeneration system to the coverglass, we observed some tethers with an apparent BM increase followed by tether disappearance (Figure 3A). On average, out of ∼ 30 initial tethers in the field of view, we observed about 5 tethers (17%) that showed this kind of BM increase and tether disappearance. The increase in BM and tether disappearance suggests that *Gk*DnaC hexamers alone are capable of unwinding this 90 bp substrate. Using DNA substrates with a longer duplex region, however, did not produce a significant amount of tether disappearance, likely due to the limited processivity of the *Gk*DnaC helicase. Control experiments without ATP or without *Gk*DnaC did not render any tether disappearance in BM time-courses (data not shown). There is a recording dead time of about 20 s due to solution exchange and stage restabilization for imaging (illustrated by the gray-shaded region in Figure 3A, inset). This ∼20 s dead time does not interfere with our observation of *Gk*DnaC-mediated unwinding process, since the bead BM remains constant for at least >100 seconds before the unwinding occurs. Only the beads with increasing BM pattern prior to dissociation are included in unwinding velocity analysis. For these tethers, the increasing BM time-courses can be fitted nicely by a linear line, where the slope of the fit returns the unwinding velocity of individual helicase molecule. The dwell time between the start of BM increase and the maximum BM is the time required to unwind the 90 bp duplex, and also allows the calculation of unwinding velocities. Both methods (slope in linear fits and dwell time) return with similar unwinding velocities. The average unwinding velocity of wild-type *Gk*DnaC helicase was 3.58±1.62 bp/s (N = 10) in this nearly force-free experiment. This unwinding velocity is slower than that of other ring-shape helicases determined so far, such as bacteriophage T4 gp41 (∼30 bp/s, the value estimated from extrapolating to zero force), T7 gp4 (29 bp/s at 5.2 pN, 220 bp/s at 11.2 pN) and *Escherichia coli* DnaB (80∼50 bp/s, the value estimated from extrapolating to zero force) [8], [9], [10]. In some cases (<10%), there is a pause before tethers disappear, likely due to the potential reannealing of the unwound ssDNA to its complementary strand. These tethers were not included in the unwinding velocity analysis.

**Figure 4:**
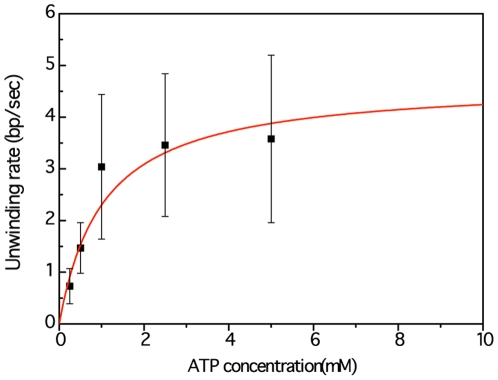
ATP dependence of unwinding velocities of *Gk*DnaC WT obtained from smTPM. Helicase unwinding velocities were measured at different ATP concentrations (0.25–5.0 mM). The data were fitted to Michaelis-Menten kinetics, yielding *K*_M, ATP_ of 1.03±0.46 mM and *V*_max_ of 4.69±2.00 bp/s.

To further confirm that the increase in BM time-course indeed reflects helicase-catalyzed unwinding processes, we measured the unwinding velocities at different ATP concentrations. Even though hexamer helicases hydrolyze a number of ribonucleoside and deoxyribonucleoside triphosphates (rNTPs and dNTPs) in the absence of DNA substrates, their unwinding efficiency is powered by its ATPase, and is thus ATP dependent. We measured the unwinding velocity of *Gk*DnaC at 5 different ATP concentrations, and determined its unwinding velocities, as shown in Figure 4 and Table 1. The Michaelis-Menten fit yielded values of *V*_max_ of 4.69±2.00 bp/s and *K*_M, ATP_ of 1.03±0.46 mM. The observed ATP dependence provides direct and strong evidence that the detected increase in BM time-course describes the *Gk*DnaC-catalyzed unwinding process.

**Table 1:** ATP dependence of the unwinding velocity measured by smTPM.

ATP conc. (mM)	Unwinding velocity (bp/s)
5.0	3.58±1.62 (N = 15)
2.5	3.46±1.45 (N = 10)
1.0	3.04±1.40 (N = 10)
0.5	1.47±0.57 (N = 5)
0.25	0.73±0.34 (N = 10)

### Helicase-DNA interaction is correlated with its unwinding

Our gel shift results suggested that residues located at the inside channel of the helicase that participate in ssDNA binding could affect the helicase unwinding efficiency. The enhanced helicase efficiency of the enzymes carrying a single point mutation at these sites might lead to a faster unwinding velocity. To confirm the results from the gel shift assay, we performed single-molecule time-course analyses. The increasing BM time-course was fitted to yield an unwinding velocity for each helicase molecule (Figure 3). Exemplary BM time-courses of wild-type and mutant helicases are shown in Figure 3 together with the compiled histograms supplemented with bootstrapping analysis. As shown in Figure 3C and 3D, the mean unwinding velocities of R145A/K146A and R332A mutants were found to be 10.52±2.09 bp/s (R145A/K146A, N = 15) and 7.50±2.93 bp/s (R332A, N = 12), respectively. Both mutants showed apparent, statistically significant, increased unwinding velocities as compared to the wild-type enzyme (3.58±1.62 bp/s), with the consideration of the error associated with the conversion factor. In addition, in the BM time-courses of mutants and the wild-type helicases, no apparent backstep motion was observed in the limited resolution of the TPM experiments. As summaried in Table 2, enhanced unwinding velocities (and unwinding efficiency) of these helicase mutants are not due to an increase of their ATPase activity, since their ATPase rates are basically similar to that of the wild-type enzyme. Based on kinetic evidence at the single-molecule level and structural analysis in a previous study [6], we speculated that helicase translocation and unwinding can be effectively modulated by its binding affinity towards ssDNA. This can be achieved by direct alteration of the helicase itself as shown in our mutation studies. Previous studies reported that the unwinding velocities of hexameric helicases have a strong force dependence [8], [9], [10]. Although the applied force mainly modulates the stability of DNA substrate, but the external forces may also influence the helicase-DNA interaction resulting in an efficient unwinding action.

**Table 2:** Enzymatic activities of wild-type and mutant *Gk*DnaC helicases.

	WT	R332A	R145A/K146A	R420A
Mutant locations	-	CTD, α15	NTD, α-hairpin (α7)	Arg finger
K_D_ (M)	4.69±0.40×10^−8^	6.64±0.90×10^−6^	2.70±0.60×10^−5^	n.d.^a^
Unwinding efficiency (fold)	1	3.67±0.85	2.86±0.54	No activity
ATPase activity (µM/min)	32.58±10.49	31.80±15.41	33.95±6.58	No activity
Unwinding velocity (bp/s)	3.58±1.62	7.50±2.93	10.52±2.09	n.d.^a^

a n.d., not detected.

Based on structural analysis [6], we identified that Arg145 interacts with the ribose of nucleotide through hydrogen bonding, whereas Lys146 binds in the same manner to the base of nucleotide. In the CTD (C-terminal domain) collar, Arg332 is located in the DNA-binding Loop I which is structurally conserved in RecA-like family [6], [24]. The Loop I (A) and Loop I (B) from both subunits buttress the 3′-end of ssDNA, and these flanking loops function like a clamp to mediate the ssDNA binding via hydrogen bonds and a salt-bridge to the phosphate backbone. Because that hydrogen bonding interaction plays a critical role between protein and DNA, changing these positively charged residues (Arg145, Lys146 and Arg332) to alanine alters the protein-DNA interaction significantly. In addition, previous studies showed that the *E. coli* DnaB hexamer could occlude 20-mer ssDNA in length, but only 10-mer ssDNA was strongly protected against nuclease digestion [25], [26], [27]. We also only observed 9-mer ssDNA seating on the basic DNA-binding pocket formed from two subunits in the asymmetric unit, although we used 15-mer oligo(dT) for crystallization [6]. Therefore, in *G. kaustophilus*, we suggested that the DNA-binding site we found in NTD (N-terminal domain) collar probably belongs to a “strong” DNA-binding site. However, in the cases of the papillomavirus E1 helicase and *E. coli Rho* helicase (smaller diameter in central channel of 17∼20 Å), nucleic acid is bound within the channel via loops that form a “spiral staircase” protruding from each subunit [28], [29]. We can thus speculate that two DNA-binding sites exist in the central channel of hexameric *Gk*DnaC: one located in the wider NTD collar of 50 Å in diameter (“strong” DNA-binding site) and the other in the narrow CTD collar of ∼20 Å (“weak” DNA-binding site). Two DNA-binding sites independently bind and release DNA in response to the signals received from the NTPase site upon translocation. The “strong” DNA-binding site that tightly binds nucleic acids via hydrogen bonding is responsible for stabilization of the DNA strand, so it could optimize processivity and also provide a stable DNA template for priming. On the other hand, the “weak” DNA-binding that transiently binds nucleic acids via loops protruding from each subunit is responsible for the directional motion forward to the junction. Consequently, reducing the interaction between NTD of protein and DNA is a potential way of loosening DNA strand, which in turn might increase helicase translocation velocity.

### Primase accelerates the unwinding velocity of *Gk*DnaC helicase

The chromosomal DNA replicases are multiprotein molecular machines. Previous studies showed that a ring-shaped hexameric DNA helicase forms a complex with either its loader or primase, resulting in the alteration of enzymatic activity [12], [30], [31]. To clarify how cooperativity and functional relevance between DnaC helicase and primosomal proteins in *G. kaustophilus* are achieved, we determined the duplex unwinding and ATP hydrolysis activities when DnaC helicase is in complex with DnaG or DnaI. Previous studies have shown that in the absence of nucleotides, *Gk*DnaC and *Gk*DnaI can form a stable complex, which facilitates ssDNA binding [12]. Here, we also observed that *Gk*DnaC helicase forms a stable complex with *Gk*DnaG primase in the presence of ATP using native PAGE and gel filtration (data not shown). As shown in Figure S6A, the unwinding activity and ATPase activity of the *Gk*DnaC helicase/*Gk*DnaG primase complex were increased 1.5- and 2-fold, respectively, as compared to those of *Gk*DnaC alone. By contrast, helicase unwinding was inhibited when *Gk*DnaC was pre-incubated with equimolar amounts of *Gk*DnaI loader prior to the assay. The *Gk*DnaC-*Gk*DnaI complex exhibited a lowered ATP hydrolysis rate (18.1±5.4 µM/min), almost half of that exhibited by *Gk*DnaC alone (32.6±10.5 µM/min). We noted that *Gk*DnaI has no detectable ATPase activity by itself under identical assay conditions (Figure S6B). It is likely that the binding of DnaI to DnaC may induce a conformational change in the ATP-binding pocket of DnaC helicase, resulting in lower ATP hydrolysis and unwinding efficiency.

Consistent with previous studies [31], [32] and as mentioned above, our bulk assay showed increases both in helicase activity and in ATPase activity of *Gk*DnaC in the presence of *Gk*DnaG primase, while reduced helicase activity was found in the presence of loader protein *Gk*DnaI (Table 3). However, there are several factors that can lead to enhanced helicase efficiency, such as enhanced helicase processivity and enhanced helicase unwinding velocity. In addition, there is no existing model to explain why primase can stimulate helicase activity. Thus, we also measured the unwinding velocities of *Gk*DnaC-*Gk*DnaG and *Gk*DnaC-*Gk*DnaI complexes using smTPM in real-time (Figure 3B). The unwinding velocity of *Gk*DnaC-*Gk*DnaG was 2-fold increased to 7.44±3.41 bp/s (N = 18), while no apparent unwinding process was observed for the *Gk*DnaC-*Gk*DnaI complex within 30 minutes of recording (data not shown). Due to our design of nicked substrates, the disappearance of tethers signals the completion of the unwinding process. Therefore, the number of tethers retained at a given reaction time denotes the progress of the unwinding process. Although no unwinding velocity was determined for *Gk*DnaC-*Gk*DnaI, we compared the unwinding activity of *Gk*DnaC and its complexes by measuring the number of tethers retained in the same field-of-view at various reaction time, and presented by the percentage of disappeared bead in Figure 3E. Due to the substrate design of the single-molecule experiments, the time-course of disappeared beads directly reflects the helicase unwinding activity. For the *Gk*DnaC-*Gk*DnaI complex, the percentage of disappeared bead is lower than that of *Gk*DnaC alone, and much lower than that of the *Gk*DnaC-*Gk*DnaG complex. It is likely that tight binding between the *Gk*DnaC-*Gk*DnaI complex and ssDNA (K_D_  = 47.3 nM, [12]) serves as a roadblock, and makes it unfavorable for DnaC helicase to translocate on the tracking strand, the strand that helicase engages and translocates along. Our studies show that *Gk*DnaG primase accelerates the rate of ATP hydrolysis of helicase resulting in faster unwinding velocity. It is possible that primase enhances the helicase unwinding velocity by increasing ATPase activity and/or by inducing a conformational change that favors rapid movement on the tracking strand. Previously report also indicated that primase can enhance the processivity of the helicase by stabilizing DNA binding and hexamer formation when it associates with the helicase [33]. The observation that the unwinding velocity of helicase could be modulated by the efficiency of ATP hydrolysis but not interaction between the tracking strand and helicase in the case of the primosomal protein, is in strong contrast to the helicase mutagenesis studies.

**Table 3:** Enzymatic activities of *Gk*DnaC, *Gk*DnaC-*Gk*DnaG complex and *Gk*DnaC-*Gk*DnaI complex.

	*Gk*DnaC	*Gk*DnaC-*Gk*DnaG	*Gk*DnaC-*Gk*DnaI
	helicase	helicase-primase	helicase-loader
Unwinding efficiency (fold)	1	1.45±0.21	0.15±0.07
ATPase activity (µM/min)	32.58±10.49	79.51±18.21	18.08±5.44
Unwinding velocity (bp/s)	3.58±1.62	7.44±3.41	_^a^

a The unwinding action is inhibited when *Gk*DnaC helicase is bound to its loader *Gk*DnaI.

In summary, our ensemble-averaged and single-molecule studies on *Gk*DnaC helicase and its mutants suggest that how helicase interacts with its tracking DNA strand directly affects the unwinding process. Reducing the DNA-binding affinity of the residues located inside the central channel of the *Gk*DnaC helicase efficiently enhances the unwinding efficiency and unwinding velocity without affecting the ATPase activity. Therefore, we suggested that the ring-shaped hexameric helicase might have a variety of conformations, and it can unwind more efficiently by a special conformation which is favorable for speedy translocation with a weaker ssDNA-binding ability. As expected, replication accessory proteins, such as *Gk*DnaG primase, stimulated helicase activity. However, different from the helicase mutants, the stimulation is caused because that primase could help to regulate ATPase rate of the helicase achieving a rapider unwinding velocity. These two different modes of helicase activity modulation demonstrate that both protein-DNA interaction and protein-protein interaction regulate the action of the replicative helicase. Apparently, however, the enhanced ATPase and helicase unwinding velocity are not sufficient to attain the replisome rate required *in vivo*. Other accessory proteins are thus essential to interact with *Gk*DnaC helicase for further enhancement of its unwinding velocity and processivity. Future studies of the helicase activity of *Gk*DnaC in complex with individual and sets of accessory proteins as well as that of the holoenzyme will elucidate the complexity of the regulatory network within the whole replisome machinery.

## Supporting Information

Figure S1 **The rate of ATP hydrolysis of** ***Gk*** **DnaC WT in the presence of different length of ssDNA.** Reactions were initiated by addition of *Gk*DnaC WT (1 µM), and the rate of ATP hydrolysis was monitored by following NADH oxidation at 340 nm. The experiments were performed using different length of ssDNA (15-mer, 30-mer and 70-mer). Except 70-mer ssDNA with random sequence, the ssDNA we used here all belong to single-stranded oligo-dT DNA (50 nM).
(TIF)

Figure S2 **The initial unwinding time point determination by 1^st^ derivative.** **A–D.** The raw data of helicase unwinding fork-AC90 DNA substrates, in the present of *Gk*DnaC wild-type, *Gk*DnaC+*Gk*DnaG, *Gk*DnaC mutant R332A and R145K146A, respectively. There is a recording dead time of about 20 s due to solution exchange and stage restabilization for imaging (shaded area). **E–H.** The 1^st^ derivative of the unwinding trace returns the initial unwinding time point. The solid red line represents the mean of the derivatives (which suppose to be zero). The dashed lines show the 95% marginal bond of the derivatives. It shows that after 270 sec, the derivatives grow over the bonds which indicate that the slope of the raw trace leaves zero at the time, that we determine as initial unwinding time point (red circle).
(TIF)

Figure S3 **Sequence alignment.** The schematic diagram showed the sequence alignment of DnaB-like helicases from *G. kaustophilus* (*Gk*DnaC), *B. stearothermophilus* (*Bst*DnaB), *Thermus aquaticus* (*Taq*DnaB), and *E. coli* (*Eco*DnaB) that labeled with residue numbers relative to that of *Gk*DnaC. Residues that are completely conserved, identical and similar among family members are shaded in green, yellow and cyan, respectively. The asterisk showed the important residues (R45, R117, R120, R145, K146, K309, R330, R332, R344 and R420) that influenced DNA-bound.
(TIF)

Figure S4 **Gel shift assay of increasing concentrations of** ***Gk*** **DnaC WT.** The DNA unwinding activities of protein were measured by monitoring the amount of unwound ssDNA product. The reaction was carried out in the presence of increasing amounts of purified proteins as indicated (0.1, 0.15, 0.25, 0.5, 1, 2 and 4 µM). To normalize these values, the percentage of product was calculated using the equation, %Unwound  = (%U_S_ **–**%U_0_)/(%U_100°C_ **–**%U_0_) (detail in materials and methods). Two independent experiments are shown here and are represented as red and blue lines (right panel).
(TIF)

Figure S5 **The rate of ATP hydrolysis of** ***Gk*** **DnaC under different molar ratio of protein to ssDNA.** The rate of ATP hydrolysis is proportional to the rate of the decrease in absorbance at OD_340_, and it can be calculated according to the formula: ΔA_340_/time (s^−1^) ×9820 =  rate of ATPase (µM/min). Reactions were initiated by the addition of 1 µM *Gk*DnaC WT (per monomer) into the mixture in the presence of 15-mer single-stranded oligo-dT DNA (50 nM or 10 µM). Two independent experiments are shown here in different molar ratio system.
(TIF)

Figure S6 **Unwinding and ATPase activity of** ***Gk*** **DnaC were affected when** ***Gk*** **DnaC was associated with primosomal protein.** **A.** The effect of primosomal protein on helicase unwinding efficiency. The abscissa shows varying enzyme and complex formation, which is correlated to the unwinding efficiency (fold) shown on the top of each bar. **B.** The effect of primosomal protein on ATP hydrolysis of *Gk*DnaC. The rate of ATP hydrolysis was calculated from the rate of change in absorbance at 340 nm due to oxidation of NADH. Data represent the average of three independent experiments.
(TIF)
