# Correction: Mutations Altering the Interplay between *Gk*DnaC Helicase and DNA Reveal an Insight into Helicase Unwinding

**DOI:** 10.1371/annotation/24720869-8a5d-44c1-bcaa-2f2138e8c8b7

**Published:** 2012-01-12

**Authors:** Yu-Hua Lo, Shih-Wei Liu, Yuh-Ju Sun, Hung-Wen Li, Chwan-Deng Hsiao

The name of the fourth author contained an error in the byline of the PDF version. The correct name is Hung-Wen Li.

