# Supplementary figures and images for: Mutations Altering the Interplay between GkDnaC Helicase and DNA Reveal an Insight into Helicase Unwinding
Source: PLoS One. 2011 Dec 13;6(12):e29016. doi: 10.1371/journal.pone.0029016 (PMC3236778; doi:10.1371/journal.pone.0029016)

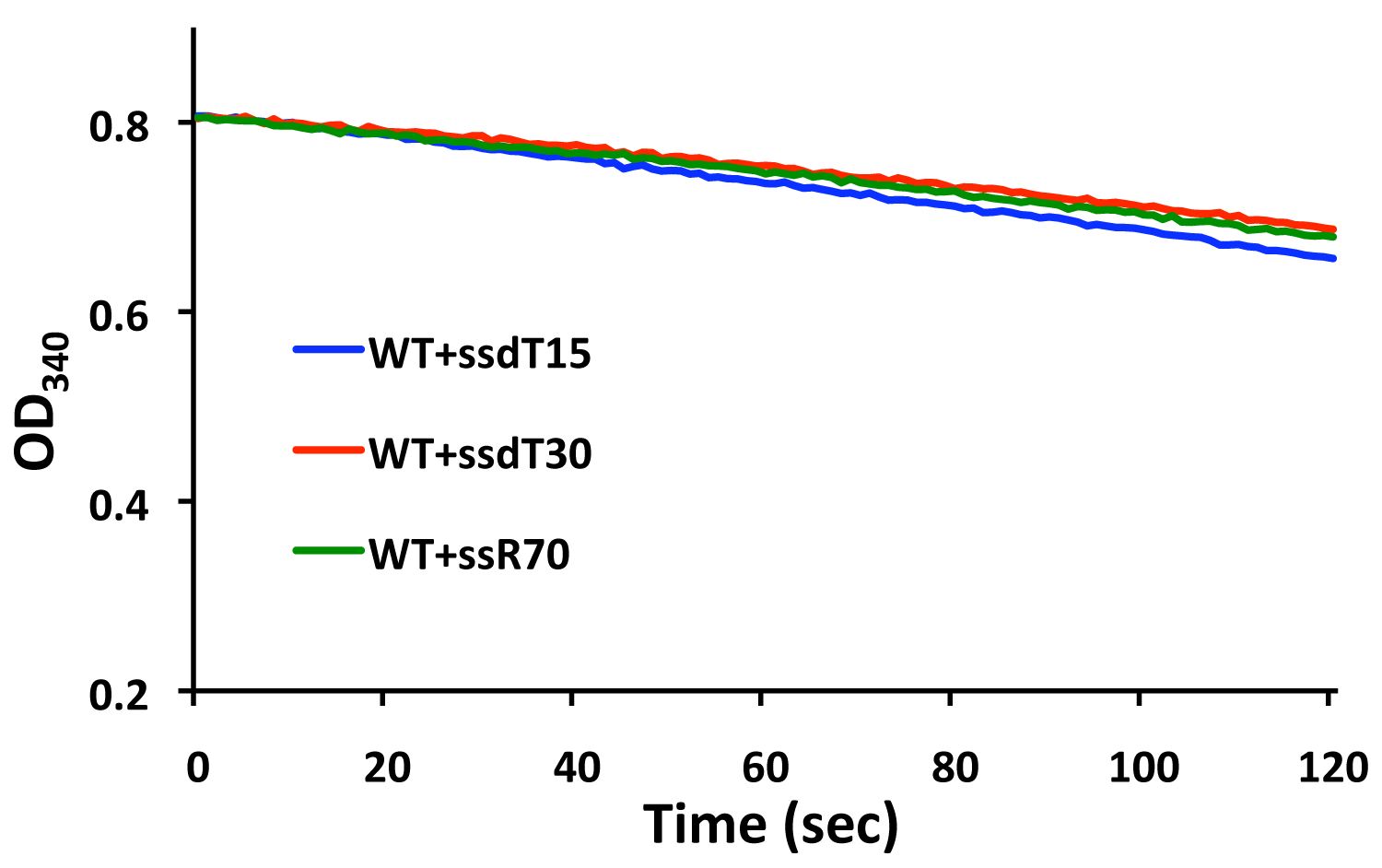

Supplement: Figure S1 — The rate of ATP hydrolysis of Gk DnaC WT in the presence of different length of ssDNA. Reactions were initiated by addition of GkDnaC WT (1 µM), and the rate of ATP hydrolysis was monitored by following NADH oxidation at 340 nm. The experiments were performed using different length of ssDNA (15-mer, 30-mer and 70-mer). Except 70-mer ssDNA with random sequence, the ssDNA we used here all belong to single-stranded oligo-dT DNA (50 nM). (TIF) [file pone.0029016.s001.tif]

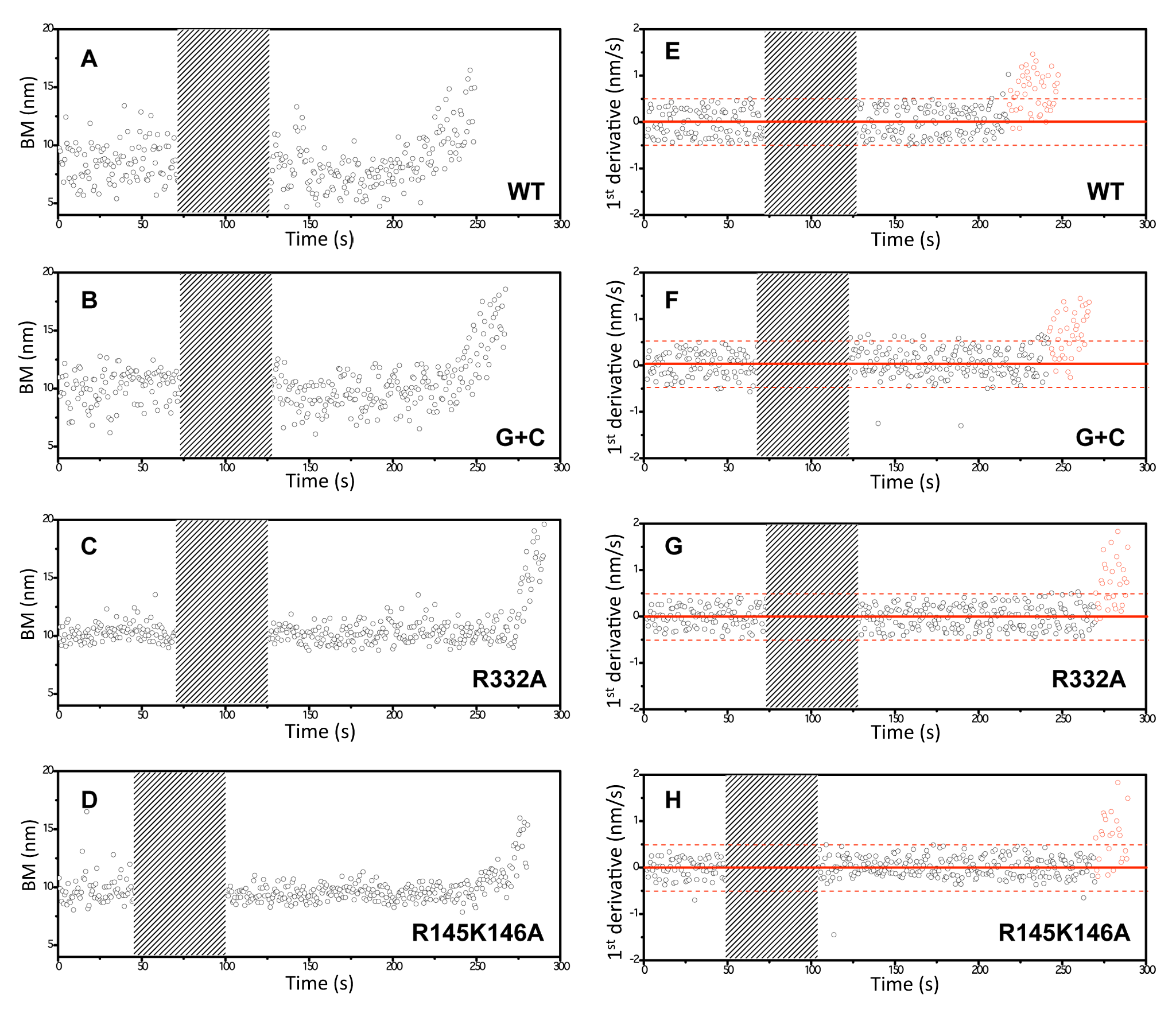

Supplement: Figure S2 — The initial unwinding time point determination by 1st derivative. A–D. The raw data of helicase unwinding fork-AC90 DNA substrates, in the present of GkDnaC wild-type, GkDnaC+GkDnaG, GkDnaC mutant R332A and R145K146A, respectively. There is a recording dead time of about 20 s due to solution exchange and stage restabilization for imaging (shaded area). E–H. The 1st derivative of the unwinding trace returns the initial unwinding time point. The solid red line represents the mean of the derivatives (which suppose to be zero). The dashed lines show the 95% marginal bond of the derivatives. It shows that after 270 sec, the derivatives grow over the bonds which indicate that the slope of the raw trace leaves zero at the time, that we determine as initial unwinding time point (red circle). (TIF) [file pone.0029016.s002.tif]

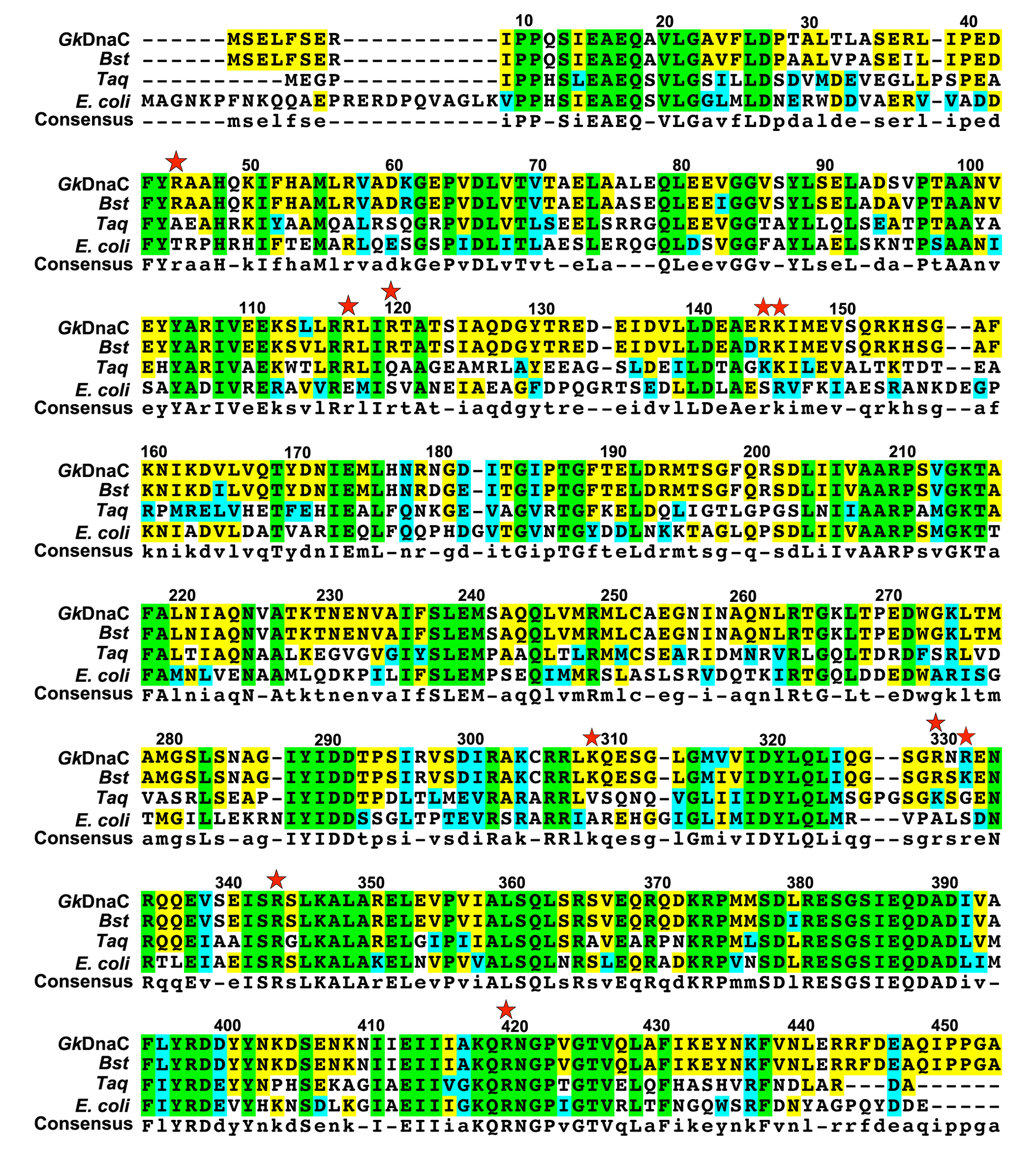

Supplement: Figure S3 — Sequence alignment. The schematic diagram showed the sequence alignment of DnaB-like helicases from G. kaustophilus (GkDnaC), B. stearothermophilus (BstDnaB), Thermus aquaticus (TaqDnaB), and E. coli (EcoDnaB) that labeled with residue numbers relative to that of GkDnaC. Residues that are completely conserved, identical and similar among family members are shaded in green, yellow and cyan, respectively. The asterisk showed the important residues (R45, R117, R120, R145, K146, K309, R330, R332, R344 and R420) that influenced DNA-bound. (TIF) [file pone.0029016.s003.tif]

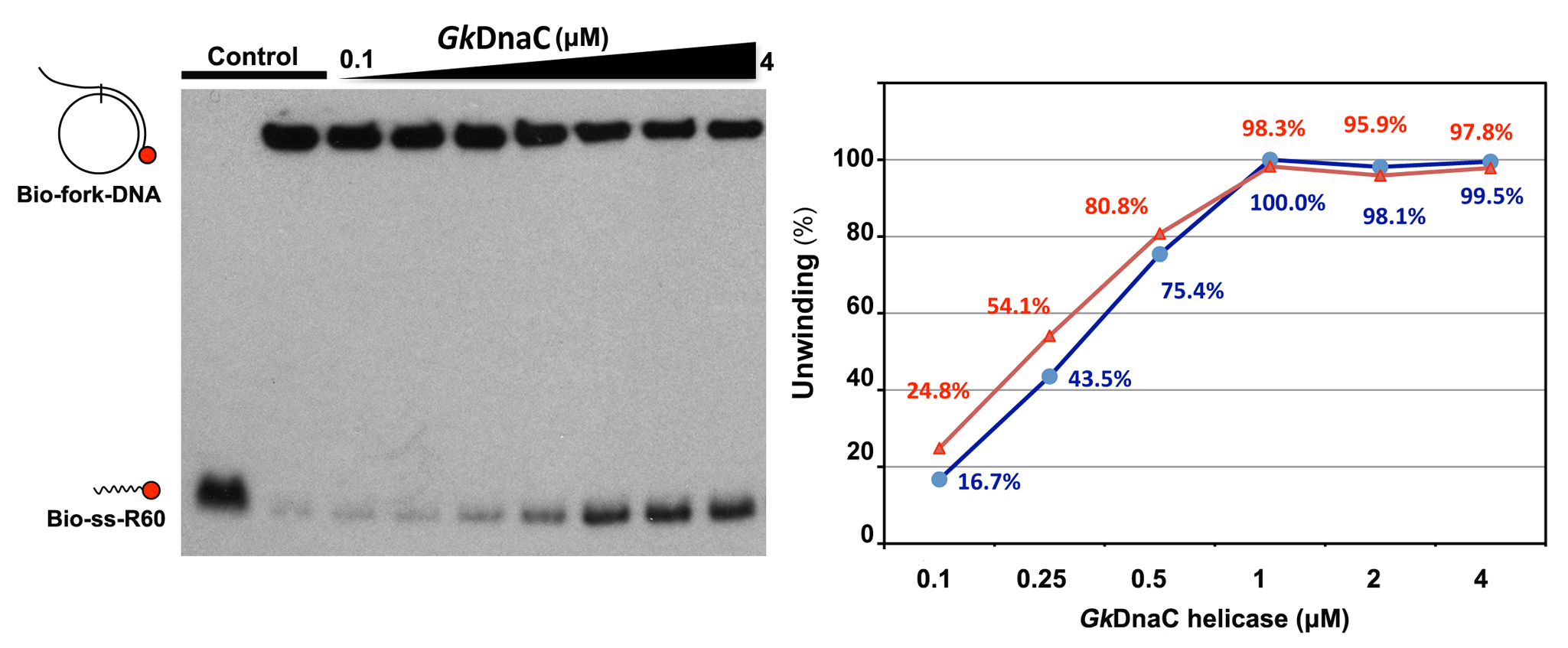

Supplement: Figure S4 — Gel shift assay of increasing concentrations of Gk DnaC WT. The DNA unwinding activities of protein were measured by monitoring the amount of unwound ssDNA product. The reaction was carried out in the presence of increasing amounts of purified proteins as indicated (0.1, 0.15, 0.25, 0.5, 1, 2 and 4 µM). To normalize these values, the percentage of product was calculated using the equation, %Unwound = (%US –%U0)/(%U100°C –%U0) (detail in materials and methods). Two independent experiments are shown here and are represented as red and blue lines (right panel). (TIF) [file pone.0029016.s004.tif]

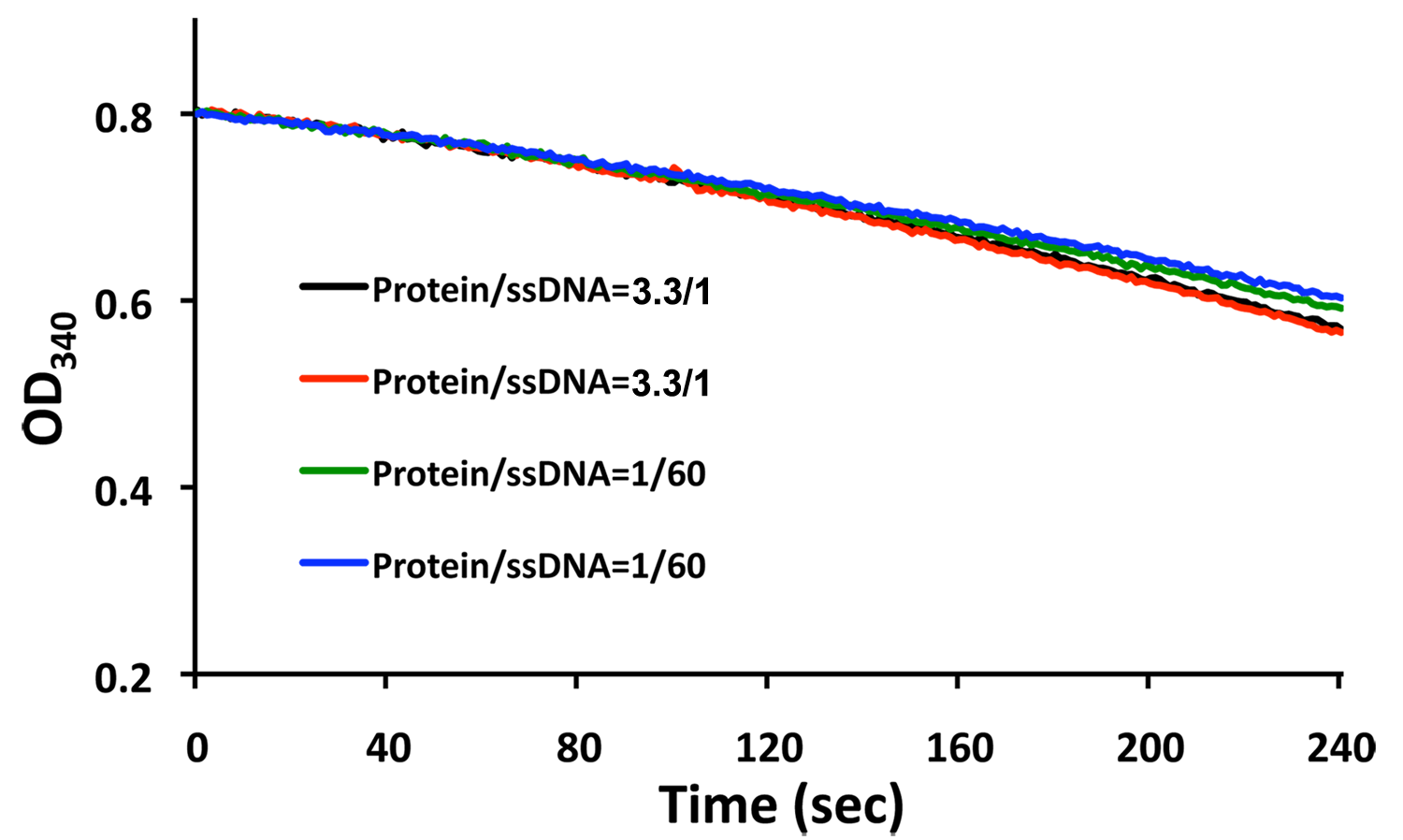

Supplement: Figure S5 — The rate of ATP hydrolysis of Gk DnaC under different molar ratio of protein to ssDNA. The rate of ATP hydrolysis is proportional to the rate of the decrease in absorbance at OD340, and it can be calculated according to the formula: ΔA340/time (s−1) ×9820 = rate of ATPase (µM/min). Reactions were initiated by the addition of 1 µM GkDnaC WT (per monomer) into the mixture in the presence of 15-mer single-stranded oligo-dT DNA (50 nM or 10 µM). Two independent experiments are shown here in different molar ratio system. (TIF) [file pone.0029016.s005.tif]

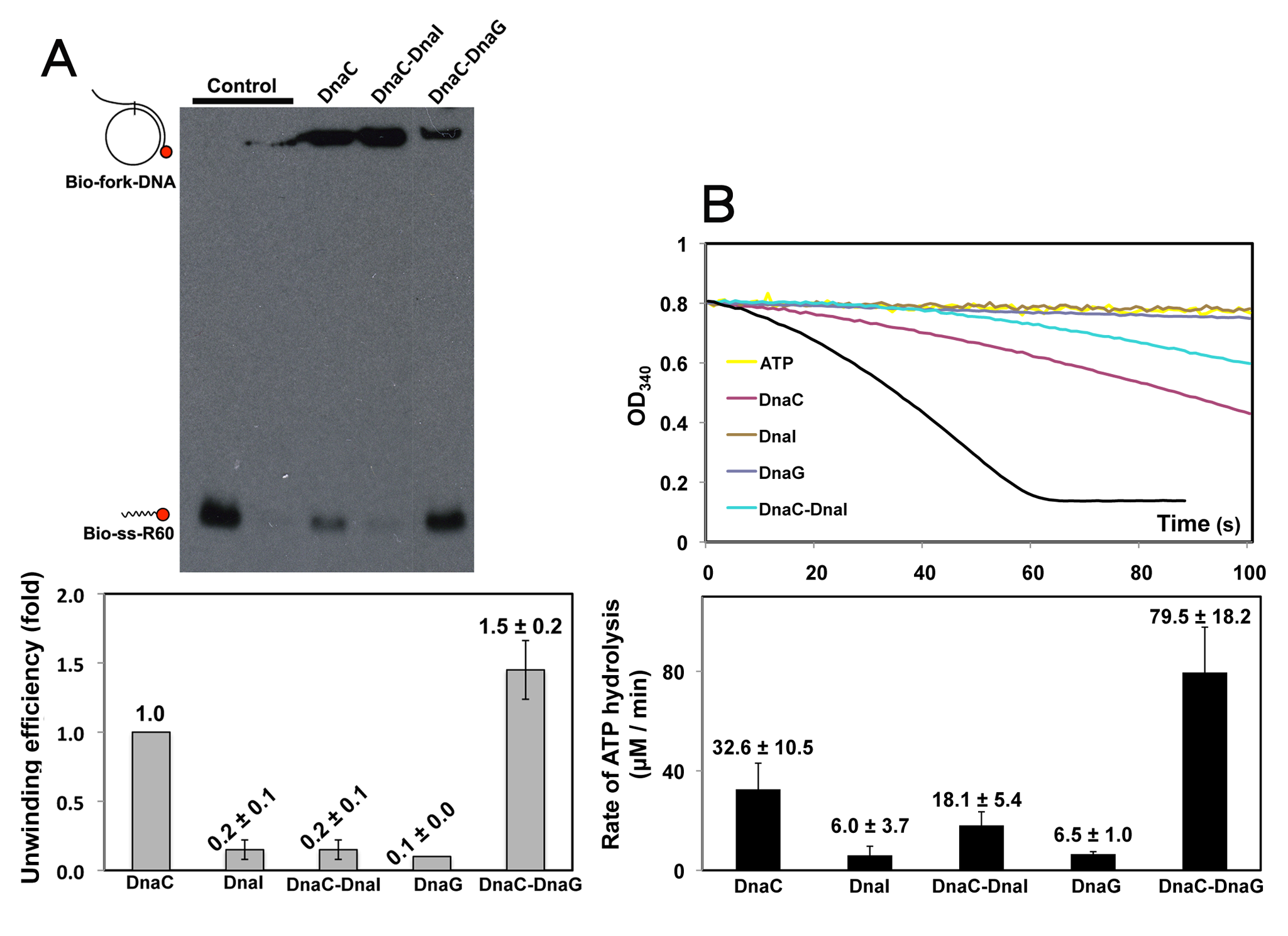

Supplement: Figure S6 — Unwinding and ATPase activity of Gk DnaC were affected when Gk DnaC was associated with primosomal protein. A. The effect of primosomal protein on helicase unwinding efficiency. The abscissa shows varying enzyme and complex formation, which is correlated to the unwinding efficiency (fold) shown on the top of each bar. B. The effect of primosomal protein on ATP hydrolysis of GkDnaC. The rate of ATP hydrolysis was calculated from the rate of change in absorbance at 340 nm due to oxidation of NADH. Data represent the average of three independent experiments. (TIF) [file pone.0029016.s006.tif]
